# A Hybrid Oral Rehabilitation of Hypohidrotic Ectodermal Dysplasia: A Conservative Approach with Three-Year Follow-Up

**DOI:** 10.1155/2021/7469595

**Published:** 2021-12-11

**Authors:** Aryen Kaushik, HarshVardhan Sinha, M. N. Hombesh, Pooja Rani, Taranjeet Kaur

**Affiliations:** ^1^Department of Prosthodontics, ITS Dental College, Greater Noida, India; ^2^Department of Prosthodontics, Saraswati Dental College, Lucknow, India; ^3^Department of Prosthodontics, College of Dental Sciences, Davangere, India; ^4^Department of Prosthodontics, Buddha Institute of Dental Sciences and Hospital, Patna, India

## Abstract

This case report presents a 19-year-old male patient with hypohidrotic ectodermal dysplasia, having a chief complaint of multiple missing teeth. Atraumatic extraction of the teeth with hopeless prognosis was done, and teeth with grade 2 mobility were submerged using cast dowel and coping. Following this, incremental increase in the vertical dimension was made using removable flexible splint of two-millimeter thickness. After facebow transfer and making appropriate eccentric bite records to program the semiadjustable articulator, wax-up was done at the desired vertical dimension (VD). The upper arch was finally restored using a long-span fixed partial denture and lower arch using bilateral attachment (Rhein 83) retained cast removable partial denture as a definitive prosthesis. Therefore, in conditions like hypodontia or oligodontia caused due to ectodermal dysplasia, attachment retained removable partial denture may prove beneficial by effectively distributing the occlusal forces. In clinical scenarios where implant is not feasible or not opted by the patient, this combination treatment may be a viable option.

## 1. Introduction

Ectodermal dysplasias (EDs) are a heterogeneous group of rare congenital disorders (incidence at birth-1 in 100,000 males), which involve primary developmental defects of two or more tissues derived from the ectoderm like skin, nails, teeth, and eccrine glands. The disorder can be classified as hidrotic type (Clouston syndrome) inherited as autosomal dominant condition and hypohidrotic or anhidrotic type (Christ-Siemens-Touraine syndrome) which is inherited as X linked recessive attribute [1]. Although the National Foundation for Ectodermal Dysplasia has listed twenty different types of such disorders, severity may differ within the same type of ED [1]. In these cases, the prosthodontic interventions mainly focus to enhance the esthetics, phonetics, and function, by various fixed or removable treatment approaches [1–4].

This specific case was unique from the previous case studies reported as the patient had hyposalivation and severe gag reflex and presented with mixed dentition, posing a problem in treatment planning. Moreover, there was a marked loss in occlusal vertical dimension. Although every treatment option has its own advantages and disadvantages, we have tried to manage this case using a hybrid approach by rehabilitating the upper arch with a long-span fixed partial denture and lower arch with bilateral attachment retained removable partial denture, a treatment approach which has not been documented in previous case report literature.

## 2. Case Presentation

**Figure 1:**
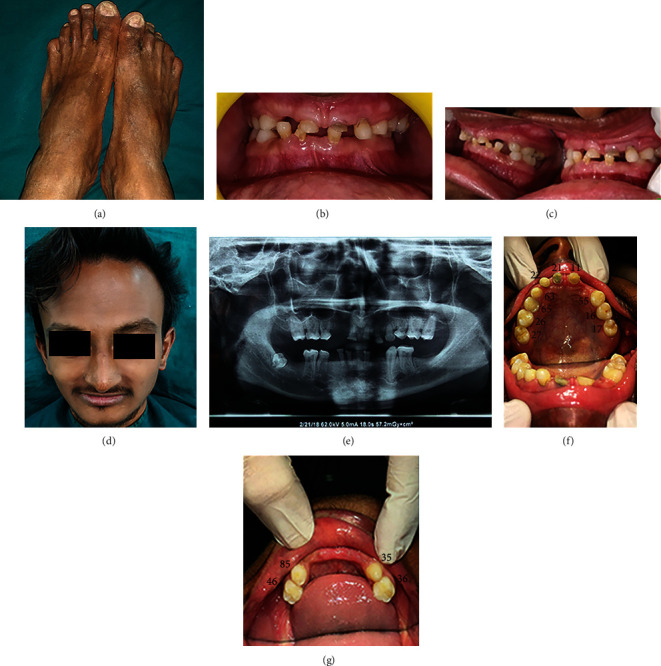
(a) Dystrophy of toe nails, (b–d) pretreatment occlusal vertical dimension loss, (e) pretreatment orthopantomogram, (f) pretreatment maxillary, and (g) mandibular arch.

A 19-year-old male with a chief complaint of multiple missing teeth and difficulty in chewing food was referred to the Department of Prosthodontics by his physician. On extraoral clinical examination, he had frontal bossing, depressed nasal bridge, thick everted lower lips, sparse facial hair, sunken cheeks, and dystrophy of toe nails. The patient was concerned regarding his esthetics and seemed to have a low self-esteem. Previous health reports had mentioned the diagnosis as hypohidrotic type of ectodermal dysplasia. Extraoral examination revealed sunken cheeks, deep nasolabial fold, mild frontal bossing, thin sparse facial hair, and normal nails (Figure 1(a)). Various treatment modalities like direct composite restorations, fixed partial dentures, and removable prosthetic options like overdentures, endosseous implants, mini-implants, and zygomatic implants have been advocated by various authors [1–4].

Intraoral examination revealed dry oral mucosa (later confirmed by sialometry test) and residual ridge with adequate width and height. The teeth present were 17, 16, 55, 11, 21, 22, 63, 65, 26, 27, 71, 81, 82, 36, 35, 85, and 46 (Figures 1(b)–1(g)). A thorough oral prophylaxis was done, and the patient was recalled after 2 weeks to assess the periodontal prognosis of each tooth. Bearing in mind the existing crown root ratio and number of teeth which would remain after extraction of grade three mobile teeth, a single unit long-span porcelain fused to metal fixed partial denture was planned for the maxillary arch. After assessing the periodontal health of the lower teeth and crown height space of 6.5 millimeters (mm), a bilateral attachment retained cast removable partial denture was planned for lower arch.

Vertical dimension (VD) of face was assessed using phonetics along with the Niswonger's physiologic method. This step was done by placing an adhesive tape in the shape of a small equilateral triangle at the tip of nose and chin and then asking the patient to repeat words ending with sound “m.” At least six to seven readings were recorded between the two points, and an average of all was considered as vertical dimension at rest. The patient was then asked to occlude all teeth, and the distance between two points revealed the vertical dimension at occlusion. Subtracting the latter from former resulted in a freeway space of seven to eight millimeters (with normal range of three to four millimeters), which was evident clinically in premolar region, indicating a loss in occlusal vertical dimension.

**Figure 2:**
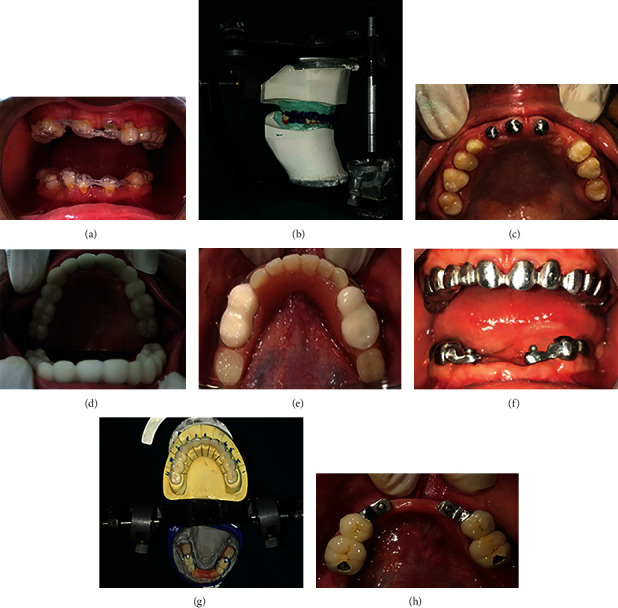
(a) Flexible occlusal splint, (b) final wax-up at desired vertical dimension, (c) minimal tooth preparation with polished metal copings, (d) maxillary and (e) mandibular provisional restorations, (f) metal coping try-in, (g) ceramic layering of prosthesis, and Rhein 83 attachment.

An incremental increase in VD up to 4 mm over a period of 3 months was planned to assess the physiologic comfort and ensure an adequate adaptation time for temporomandibular joint [5]. Therefore, conforming to the “two splint concept,” a removable flexible splint of 2 mm thickness was worn by the patient in mandibular arch for initial 6 weeks and in the maxilla for next 6 weeks (Figure 2(a)) [5, 6]. Alternatively, a second mandibular splint of 4 mm thickness could have been given to the patient as a substitute for maxillary 2 mm thickness splint. Either ways, the desired VD increase of 4 mm would have been achieved. Initially, a hard splint was tried for the same procedure but later, a soft splint was used as the patient was more comfortable and compliant to it. Moreover, recent studies show that both hard and soft splints are equally effective in achieving the desired results of treatment [7, 8]. The soft splint in this case was not intimately adapted on the teeth with grade 2 mobility, to prevent any further detrimental effect due to occlusal forces.

Teeth with grade 3 mobility and poor crown root ratio, i.e., 71, 81, and 82, were extracted. Following 2 weeks of socket healing, diagnostic impressions were made using irreversible hydrocolloid and casts were poured. Upper diagnostic cast was mounted on Hanau wide-vue articulator using Hanau springbow transfer. Centric jaw relation record was obtained by guiding patient's jaw as per bimanual manipulation method and using 4.5 mm thickness of warm Aluwax for recording the cusp indentations (Aluwax Dental Products Co), followed by mounting of the mandibular casts. A complete tentative wax-up was performed on diagnostic mounting and putty index (Dentsply Aquasil) were made (Figure 2(b)). Teeth with inadequate crown surface area, i.e., 11, 21, and 22, were endodontically treated followed by cast post and metal coping (root submergence technique). Meanwhile, an intraoral mockup was performed using these putty indices and a provisional restorative material (Protemp4). Protrusive record (up to six millimeters) was now obtained intraorally, to program the horizontal condylar guidance on the articulator.

Conservative tooth preparation was done on maxillary teeth, using the intraoral mockup as a tooth preparation guide. As teeth were hypoplastic in nature and had a tendency to chip, only minor refinement was done using extrafine diamond bur. No occlusal reduction was attempted as increase in vertical dimension was planned (Figure 2(c)). As tapered cone shaped and malformed teeth were present, achieving the parallelism by excessive tooth reduction was not clinically challenging.

Final impressions were made using two-stage putty wash impression technique, and provisional restorations were fabricated directly using the final putty indices and luted with noneugenol temporary luting cement (3M ESPE RelyX Temp NE) (Figures 2(d) and 2(e)). Mounting of the master casts was done in centric relation at desired VD as mentioned above. Metal trial made using direct metal laser sintering technique was tried in patient's mouth to ensure marginal adaptation (Figure 2(f)). The ceramic layering with feldspathic porcelain established the occlusion plane using the final wax-up as a reference (Figure 2(g)). In this, case ceramic baked over direct metal laser sintered coping was used instead of single unit zirconia bride due to various reasons. First, the patient had an average smile line (exposing slightly more than 75% of central incisors), hence minimizing the probability of slight metal discolored margin visibility in future. Second, the retrievability of resin cement luted single unit zirconia bridge would have been challenging. Third, due to the milling process used for fabricating the metal coping substructure, the 2 mm thickness of porcelain layering could be easily standardized, hence resulting in more predictive metal porcelain bond. Fourth, it was more cost-effective for the patient when discussed.

**Figure 3:**
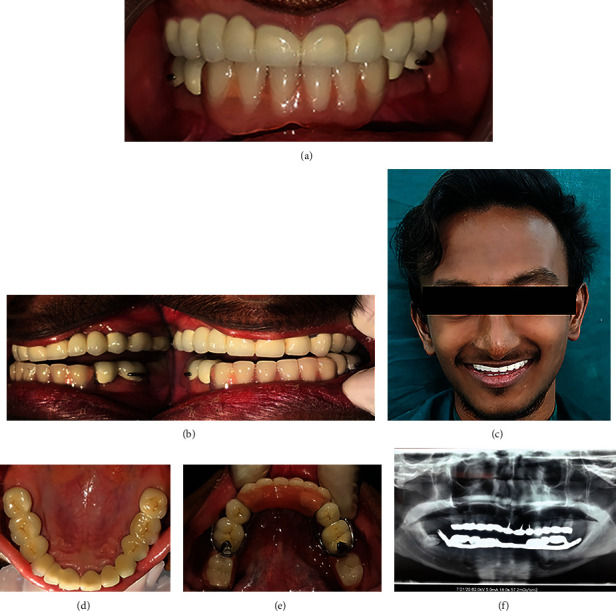
(a) Final prosthesis in maximum intercuspation, (b) group function occlusion on lateral excursion movement. (c) Posttreatment esthetics, (d, e) 3-year periodic review intraoral photographs, and (f) follow-up orthopantomogram.

A mandibular cast removable partial denture framework (Co-Cr alloy) was then designed over Rhein 83 attachment using yellow extrasoft nylon retentive cap, and passive circumferential clasps were given (Figures 2(g) and 2(h)). Occlusal contacts in maximum intercuspation position were checked before luting the fixed restoration (Figure 2(a)). Finally, the luting of the fixed partial denture using glass ionomer cement (GC Gold Label 1 Luting & Lining Cement) and final denture insertion was done after ensuring Christensen's phenomenon as well as bilateral group function occlusion intraorally (Figure 2(b)). The esthetics and phonetics of the patient were verified (Figure 3(c)).

As a part of maintenance therapy for hyposalivation, a lemon-flavored synthetic saliva spray (Glandosane Synthetic saliva spray by Fresenius Kabi Oncology Pvt. Ltd) was used by the patient four to five times a day. This would ensure timely lubrication of the mouth, hence minimizing complications like dry and rough tongue, mouth sores, caries, and halitosis. As susceptibility to enamel caries is more in these conditions due to reduced saliva, a 1.1% sodium fluoride toothpaste (Colgate PreviDent 5000 ppm Booster Plus) was prescribed once a day. Additionally, a water flosser in pulsed mode was recommended twice a day, to enhance effective cleansability in the pontic and connector region of the fixed prosthesis.

The patient was advised for a regular follow-up every 6 months. Figures 3(d) and 3(e) depict a healthy periodontal condition and good oral hygiene maintenance at a periodic recall visit after 3 years. An orthopantomogram taken after 2.5 years showed healthy abutments with minimal alveolar changes (Figure 3(f)).

## 3. Discussion

Although occlusal stops in posterior teeth are present in this case, the teeth are peg shaped, malformed, and widely spaced. Due to the lack of tooth bud formation, the alveolar bone is minimally formed and is hypoplastic in ED cases [1]. All these factors contribute to dentoalveolar tissue deficiency and severely reduced vertical dimension of occlusion.

All possible implant treatment options were initially discussed to the patient but were denied by him due to financial restrains. Dry oral mucosa has always been associated with soreness in denture bearing tissues and patient dissatisfaction with removable oral prosthesis [9]. Therefore, considering the hyposalivation, severe gag reflex, and psychological discomfort expressed by the patient, removable prosthodontic options were precluded from treatment plan for the maxillary arch. Many authors do not advocate fixed partial dentures (FPDs) with rigid connectors, especially if the prosthesis crosses the midline as only few abutments are present in most of the cases reported and the patients were in their growth phase during the treatment [10]. However, a single unit long-span FPD was planned for the maxillary arch as sufficient healthy abutment teeth were present to support the prosthesis and the patient was in late adolescence. The growth of maxilla in males has been reported to be consistent after the age of 18 [11].

Due to the abutment conditions with respect to 11, 21, and 22, root submergence technique was applied in the maxillary anterior region, as it is a promising method of maintaining the alveolar ridge dimensions at the pontic site of an FPD especially in esthetic zones [12]. For effective cleansability, a uniform clearance of 1 mm was ensured between the modified ridge lap pontic and the metal copings.

Considering the resilient nature of the extrasoft nylon retentive cap used over Rhein 83 attachment, the occlusal forces are better distributed between the lower fixed partial denture and alveolar ridge mucosa. Hence, attributing to this “stress breaker effect,” masticatory overload on the lower fixed prosthesis is circumvented, contributing to the periodontal health of the lower abutments.

As the canine teeth were missing in both the arches, a group function occlusion was provided bilaterally, ensuring the absence of premature contacts on nonworking side. The patient has been followed up for 3 years, and so far, there have been no complications.

## 4. Conclusion

In conditions like hypodontia or oligodontia caused due to ectodermal dysplasia, attachment retained cast removable partial denture may prove beneficial by effectively distributing the occlusal forces. As evident in the follow-up orthopantomogram, although the prognosis of the rehabilitation is good, a regular follow-up is necessary to assess the intraoral force factors and ensure the comfort and longevity of the prosthesis.

### 4.1. Clinical Significance

In clinical scenarios where implant is not feasible or not opted by the patient, a combination treatment of long-span fixed partial denture and an attachment retained cast removable partial denture can prove beneficial. This case gives an excellent treatment outcome in terms of esthetics as well as function, hence improving the quality of life.
